# *Ppe.RPT/SSC-1:* from QTL mapping to a predictive KASP test for ripening time and soluble solids concentration in peach

**DOI:** 10.1038/s41598-024-51599-2

**Published:** 2024-01-17

**Authors:** Cassia da Silva Linge, Wanfang Fu, Alejandro Calle, Zena Rawandoozi, Lichun Cai, David H. Byrne, Margaret Worthington, Ksenija Gasic

**Affiliations:** 1 Department of Plant and Environmental Sciences, College of Agriculture, Forestry and Life Sciences, Clemson University, Clemson, SC 29634 USA; 2 Department of Agriculture and Environmental Sciences, University of Milan, Milan, Italy; 3 Institut de Recerca i Tecnologia Agroalimentàries (IRTA), Fruitcentre, PCiTAL, Gardeny Park, Fruitcentre Building, 25003 Lleida, Spain; 4 Department of Horticultural Sciences, Texas A&M University, College Station, TX 77843 USA; 5 Department of Horticulture, University of Arkansas System Division of Agriculture, Fayetteville, AR 72701 USA

**Keywords:** Genetic mapping, Genotyping and haplotyping, Agricultural genetics

## Abstract

Genomic regions associated with ripening time (RPT) and soluble solids concentration (SSC) were mapped using a pedigreed population including multiple F_1_ and F_2_ families from the Clemson University peach breeding program (CUPBP). RPT and SSC QTLs were consistently identified in two seasons (2011 and 2012) and the average datasets (average of two seasons). A target region spanning 10,981,971–11,298,736 bp on chromosome 4 of peach reference genome used for haplotype analysis revealed four haplotypes with significant differences in trait values among different diplotype combinations. Favorable alleles at the target region for both RPT and SSC were determined and a DNA test for predicting RPT and SSC was developed. Two Kompetitive Allele Specific PCR (KASP) assays were validated on 84 peach cultivars and 163 seedlings from the CUPBP, with only one assay (*Ppe.RPT/SSC-1*) needed to predict between early and late-season ripening cultivars and low and high SSC. These results advance our understanding of the genetic basis of RPT and SSC and facilitate selection of new peach cultivars with the desired RPT and SSC.

## Introduction

Peach [*Prunus persica* (L.) Batsch] is the third most cultivated temperate tree fruit in the world after apple and pear^1^. The United States is the fifth largest peach producer in the world with an approximate production of 730 thousand tons, behind China, Spain, Italy, and Turkey^2^. Despite high production levels, variability in fruit quality has resulted in a significant decrease in US peach consumption in the last decades^3^.

One of the main consumer complaints about peaches is the lack of sensory quality, which is mostly caused by harvesting fruits at an inadequate ripening stage^4^. The process of fruit ripening plays an important role in improving peach organoleptic quality, including increases in the soluble solids concentration (SSC) to acid ratio, softening, and aroma^5,6^. SSC, measured as °Brix, is often used to express sugar content and represents one of the main drivers affecting peach consumer acceptance^7^. The amount of total SSC in ripe fruits combined with additional active compounds, such as pectin, salts, and organic acids, significantly increases consumer satisfaction in peaches^8,9^. Medium and late season cultivars have a greater capacity to accumulate sugar compared to early season cultivars^4^. Ripening time, however, affects the farm gate value of the crop with early ripening cultivars getting a higher price despite the SSC levels^10^. Therefore, breeders aim to develop new peach cultivars tailored to specific ripening periods and with enhanced sensory characteristics that balance sensory quality and maturity at harvest to ensure consumer satisfaction and encourage consumption.

New genetic resources for peach have enabled several recent studies on genetic dissection of important traits including ripening time (RPT) and SSC. Quantitative Trait Loci (QTLs) and associated signals have been identified using different approaches such as bi-parental QTL mapping^11–14^, pedigree-based analysis (PBA)^15–17^ and genome-wide association studies^18^. In addition, genomic prediction models focusing on SSC were developed^19,20^.

A large-effect QTL on chromosome 4, discovered using a low-density linkage map, suggested a pleiotropic effect between SSC and RPT^11^. Further studies also highlighted a QTL hotspot in the same region using different genetic backgrounds and mapping approaches^15,18^. Although QTLs for several traits including SSC and RPT have been discovered, the conversion into practical breeding tools is still scarce. Trait-predictive DNA tests could be used to rapidly identify valuable parents as well as to remove inferior seedlings that do not possess a target characteristic. Thus, applying these genetic assays in breeding programs could significantly enhance the efficiency of the cultivar development process^21,22^.

In peach, rapid and accurate DNA tests for predicting bacterial spot severity in fruit and chilling requirement were recently developed^23,24^. Additional tests for peach skin blush^25^, fruit pubescence^26^, subacid^27^ and flesh color^28^ are also available. Most of the existing tests were developed using PCR-based simple sequence repeat (SSR) technology. Single nucleotide polymorphism (SNP) markers have replaced SSRs and other types of molecular markers due to the considerable cost-efficiency for genotyping and their precision, stability, and easy detectability^29–31^. Kompetitive Allele-Specific PCR (KASP) assays are affordable, rapid, and robust tools to genotype SNPs of interest and are widely used due to improved cost-effectiveness compared to other technologies^23,29,31,32^.

The objective of this work was to showcase the whole process from QTL discovery to DNA test development for routine use in peach breeding for the simultaneous prediction of RPT and SSC. First, we mapped and validated the previously discovered large effect RPT and SSC QTLs on chromosome 4 within Clemson University peach breeding germplasm using a pedigree-based analysis (PBA) approach. Subsequently, we developed and validated a rapid KASP test using the target region obtained by the QTL mapping.

## Results

### Phenotypic data

In this study, we have analyzed a pedigree-connected germplasm comprising cultivars, advanced selections, and seedlings from Clemson University peach breeding program (CUPBP), previously assembled under the RosBREED project^18,22^. A breeding population containing a total of 288 seedlings, included multiple F_1_ and F_2_ families obtained from 19 parents (Supplementary Table S1), was chosen for mapping analyses. The breeding population containing 288 seedlings from the CUPBP was evaluated for RPT and SSC over two seasons (2011–2012). The RPT ranged from 150 to 235 JD with a mean of 195 and 182 in 2011 and 2012, respectively. SSC varied from 7.4 to 19.8 °Brix with means of 12.3 and 11.7 in 2011 and 2012 (Supplementary Table S2). According to the Shapiro Wilk test, RPT did not follow a normal distribution in both seasons, whereas SSC exhibited a normal distribution only in 2012 (Supplementary Table S3). According to the non-parametric Wilcoxon signed rank test, significant differences were observed in the RPT and SSC averages between seasons with a delay in the RPT and a higher SSC mean value observed in 2011. Spearman’s rank correlation analysis demonstrated significant positive coefficients between RPT and SSC in 2011 (0.67) and 2012 (0.62). Considering the correlation between seasons, RPT had the highest coefficient (0.90) and SSC the lowest (0.41) (Supplementary Fig. S1). The narrow-sense heritability was 0.84 and 0.24 for RPT and SSC, respectively.

### QTL analysis

The pedigree-connected germplasm used in this study was genotyped with the IPSC peach 9K SNP array v1^33^. A total of 1487 informative SNPs (Supplementary Table S4) were used for QTL mapping. The physical positions of the SNPs were obtained considering the peach reference genome v2^34^. We performed the QTL analysis using a Bayesian, pedigree-based QTL analysis (PBA) implemented in FlexQTL™ using the RPT and SSC phenotypic data from 2011 (RPT_2011 and SSC_2011), 2012 (RPT_2012 and SSC_2012) as well as the average values of both seasons (RPT_Ave and SSC_Ave).

The QTL analysis identified major QTLs associated with both RPT and SSC (Table 1, Supplementary Figs. S2, S3). The RPT QTL, *qRPT_SC_4*, was consistently mapped with decisive evidence (BF > 10) on LG4 in the overlapped genetic interval of 44–51 cM in both 2011 and 2012. Peak position of the *qRPT_SC_4.1* was at 45–48 cM. The SSC QTL, *qSSC_SC_4.1,* was also consistently mapped in both seasons in a wider genetic interval (38–52 cM and 43–58 cM in 2011 and 2012, respectively) and exhibited a peak position varying from 44 to 49 cM. Overall, the *qRPT_SC_4.1* and the *qSSC_SC_4.1* were mapped in the same genetic interval forming a QTL cluster (Supplementary Fig. S3). Based on the peak position mapped in both seasons and traits, an initial genetic interval comprising eight SNPs spanning from 44 to 49 cM (physical position from 10,981,971 to 12,523,245 bp) was highlighted and subsequently used for the phenotypic variance explained (PVE). The *qRPT_SC_4.1* explained approximately 59% and 53% of the phenotypic variance in RPT 2011 and 2012, respectively, while the PVE for *qSSC_SC_4.1* ranged from 26 (2011) to 35% (2012).

**Table 1:** Summary of QTLs mapped on linkage group 4 in the Clemson University peach breeding program using FlexQTL™.

Trait	QTL name	LG	Dataset	BF	Interval(cM)	Interval (bp)	Peak (cM)	Additive	PVE
RPT	qRPT_SC_4.1	4	2011	10.2	44–51	10,981,971–13,633,831	48	18.44	59.0
	qRPT_SC_4.1	4	2012	27.2	44–50	10,981,971–12,971,285	46	16.2	53.2
	qRPT_SC_4.1	4	Ave	28.9	44–47	10,981,971–11,654,504	45	17.4	53.8
SSC	qSSC_SC_4.1	4	2011	28.7	43–58	10,760,086–17,176,059	49	1.68	25.9
	qSSC_SC_4.1	4	2012	31.7	38–52	9,617,585–13,882,450	44	1.47	34.5
	qSSC_SC_4.1	4	Ave	29.9	41–49	10,280,095–12,546,297	45	1.51	36.0

*RPT* ripening time, *SSC* soluble solids concentration, *LG* linkage group, *BF* Bayes factor, *Interval* (bp − (peach reference genome v2^34^); Additive effect; *PVE* percentage of variance explained.

Datasets using the average of two seasons for both traits (RPT_Ave and SSC_Ave) were used in an independent FlexQTL™ runs and the overlapped peak position was detected at 45 cM, explaining approximately 54% and 36% of the phenotypic variance observed in RPT and SSC, respectively (Table 2). Thus, we targeted the genetic interval from 44 to 45 cM (spanning a physical position from 10,981,971 to 11,298,736 bp) for further analysis of diplotype/genotype effects.

**Table 2:** QTL peach genotypes in ripening time (RPT) and soluble solids concentration (SSC) target region spanning 44 cM (10,981,971 bp) to 45 cM (11,298,736 bp).

SNP	Nucleotides*	Position		PVE (%)*		Haplotypes			
		Physical (bp)	Genetic (cM)	RPT	SSC	H1	H2	H3	H4
**SNP_IGA_411637**	T|G	10,981,971	44.1	27.7	16.6	T	T	T	G
SNP_IGA_412338	A|G	11,208,736	45.0	5.7	4.7	A	G	G	G
SNP_IGA_412662	A|G	11,298,736	45.0	12.5	6.6	G	A	G	A
				QTL allele		Q	Q	Q	q

SNP names, position in bp (peach reference genome v2) and cM, haplotype names. Bolded = predictive SNP.

*PVE* calculated by each marker using single linear regression analysis during the KASP test validation.

*Illumina (A|B) nucleotide designation and actual nucleotides.

To account for pleiotropic effect of RPT we also performed a QTL analysis using SSC_Ave (average of the two seasons) and the RPT as a cofactor. The QTL analysis revealed that both the BF (evidence of the presence of QTL) and PVE decreased in all three datasets containing the cofactor compared to the one where no cofactors were included (Supplementary Table S5). The dataset using no cofactor showed a BF of 29.7 and explained approximately 35% of the observed phenotypic variance. The dataset using the phenotypic average of RPT as a cofactor had the lowest BF (8.4) and PVE (20.8), while the dataset incorporating the haplotype information of the RPT QTL interval exhibited the highest BF (29.4) and PVE (27.2).

### QTL haplotype, genotype and predictive markers

**Figure 1:**
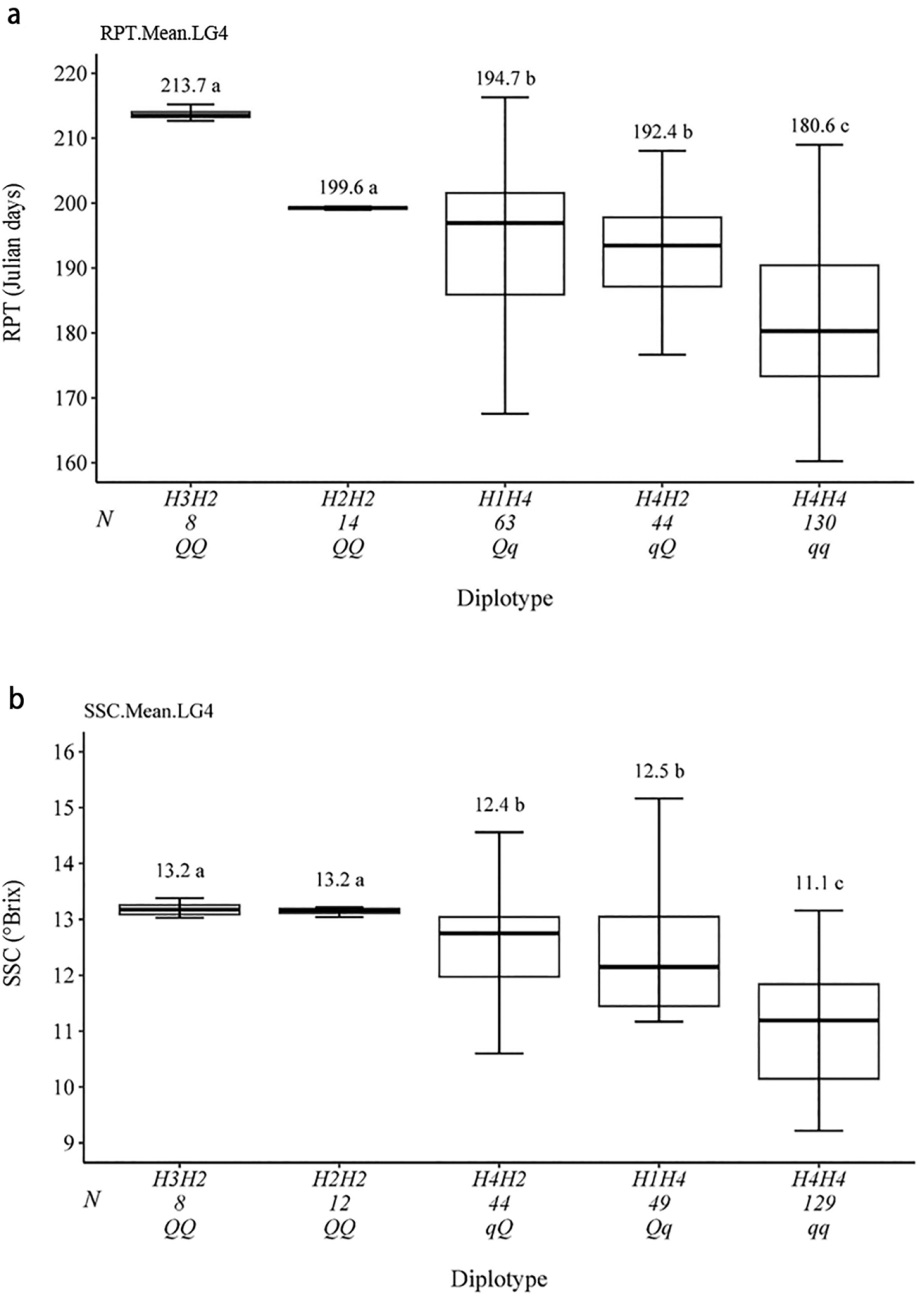
Ripening time (RPT; in Julian days, JD) (**a**), soluble solids concentration (SSC; in °Brix) (**b**) and haplotype effects in the Clemson University peach breeding material. Effects were based on diplotypes observed for *qRPT_SC_4.1* and *qSSC_SC_4.1* QTLs. Only diplotypes represented by eight or more individuals were included. N represents the number of individuals containing the phenotypic data for RPT and SSC observed in each dyplotype. Significantly different (Steele–Dwass, p < 0.05) phenotypic means are identified by different letters**.**

In this study, the haplotype analysis was carried out by selecting the SNPs within the significant QTL interval considering the RPT and SSC average datasets. Three SNPs were located within the target region of the QTL interval from 44 cM (10,981,971 bp) to 45 cM (11,298,736 bp), which was detected in the average datasets (RPT_Ave and SSC_Ave). Out of the possible six, we observed four unique haplotypes (H1–H4) defined by three SNPs within the region in the analyzed material (Table 2, Fig. 1). The sources of each haplotype are shown in the Supplementary Table S6. The haplotype H4 was associated with an early ripening, lower SSC values and was attributed to the *q* allele. The H1–H3 haplotypes were associated with increase of both traits and were assigned to the *Q* allele. The SNP_IGA_411637 was determined as the predictive marker for distinguishing the *Q* and *q* alleles (Table 2). Three statistically different QTL genotypes (*qq*, *Qq*,* QQ*) were detected in the breeding population used for QTL mapping (Fig. 1). The allele *Q* was associated with late ripening and a higher SSC and the allele *q* with early ripening and lower SSC (Fig. 1). The seedlings carrying the *QQ* genotype (H3H2 or H2H2 diplotypes) averaged 199 and 213 JD and 13.2 °Brix, while the seedlings with *Qq* genotypes (H4H2 or H1H4 diplotypes) ripened on average at 192 and 194 JD and had on average 12.4 and 12.5 °Brix. Lastly, seedlings with the *qq* genotype (H4H4) ripened on average at 180 JD and had an average of 11.1 °Brix (Fig. 1). The most frequent diplotype was H4H4 (130 seedlings phenotyped for RPT and 129 for SSC) followed by H1H4 (63 seedlings phenotyped for RPT and 49 for SSC). Seedlings carrying the *Q* allele with H2H2 (14 seedlings phenotyped for RPT and 12 for SSC) and H3H2 (8 seedlings phenotyped for RPT and SSC) diplotypes were less frequent in the population (Fig. 1).

**Figure 2:**
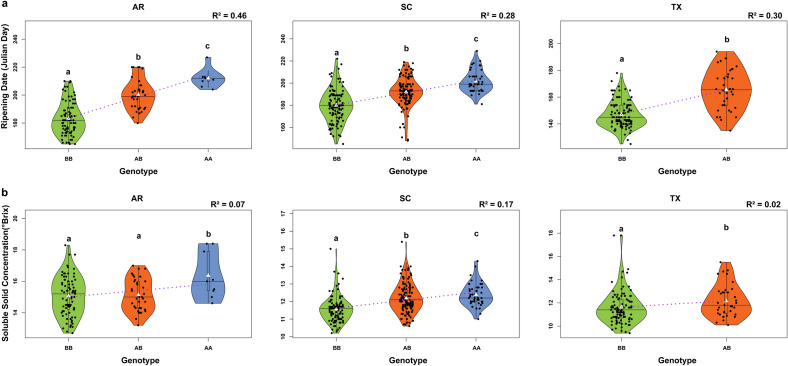
Comparison of observed ripening time (**a**) and soluble solid concentration (**b**) for the three different genotypes (AA, AB, and BB) of SNP_IGA_411637 (*Ppe.RPT/SSC_1.1*) in Arkansas (AR), South Carolina (SC) and Texas (TX) peach breeding populations. Phenotypic means that were significantly different (Tukey and Student T tests, *p* < 0.05 for three and two classes, respectively) are represented by different letters. White triangles and black horizontal lines represent mean and median phenotypic values, respectively, in each peach breeding population. R^2^ represents the proportion of variance in the dependent variable explained by the independent variable.

Although three SNPs were selected to distinguish haplotypes in the QTL region, only one, SNP_IGA_411637, was necessary to discriminate early, mid, or late-ripening individuals (Fig. 2). Single linear regression (SLR) analysis of RPT and SSC at SNP_IGA_411637 revealed 27.7 and 16.6% PVE, respectively (Table 3). Thus, to further validate our findings, we compared the RPT and SSC for the three different genotypes (AA, AB, and BB) of the SNP_IGA_411637 in Arkansas (AR), South Carolina (SC), and Texas (TX) breeding materials (Fig. 2). The comparison of the phenotypic performance of different genotypes revealed significant differences (*p* < 0.05) in all three peach breeding programs with individuals homozygous for the ‘B’ allele showing the earliest RPT and lowest SSC, and individuals homozygous for the ‘A’ allele having the latest RPT and highest SSC (Fig. 2, Supplementary Table S7). On average, individuals homozygous for the ‘B’ allele ripened 12 (SC), 16 (TX) and 17 (AR) days earlier than heterozygous individuals, and individuals homozygous for ‘A’ allele ripened 9 (SC) to 12 (AR) days later than heterozygous individuals (Supplementary Table S7). The AA genotypes were not observed in the TX material. For SSC, individuals homozygous for the ‘B’ allele had decreased SSC by 0.48 (TX) to 0.64 (SC) in comparison to the heterozygous individuals (AB). In contrast, individuals homozygous for the ‘A’ allele had increased SSC by 0.2 (SC) to 1.3 (AR) in comparison to individuals with the AB genotype (Fig. 2, Supplementary Table S7.

**Table 3:** Primer specification for Kompetitive Allele-Specific PCR (KASP). SNP target for the assay in the 9K peach SNP array, annealing temperatures (Tm, °C), and primer sequences.

Assay (SNP)	Tm	Tag	Primer sequence (5ʹ → 3ʹ)
Ppe.RPT/SSC-1.1 SNP_IGA_411637	61.6	F	*GAAGGTGACCAAGTTCATGCT*GGTTCAGTATATGGTTGTGGTTGGATGGGt
	62.0	H	*GAAGGTCGGAGTCAACGGATT*GGTTCAGTATATGGTTGTGGTTGGATGGGg
	60.4	N	CCTGTCTGGAAATCCAAGGCTGC
Ppe.RPT/SSC-1.2 SNP_IGA_412338	59.2	F	*GAAGGTGACCAAGTTCATGCT*ATCGCACCAAATTGGGTCTATGCa
	60.0	H	*GAAGGTCGGAGTCAACGGATT*ATCGCACCAAATTGGGTCTATGCg
	59.6	N	GTTCACATTGGGCACATTTGCATTTCC

FAM (F) and HEX (H) tag sequences are italicized. N = none, for the reverse primer.

^a^The SNP_IGA_412662 was unsuitable for conversion into a KASP assay and was discarded.

### KASP assay development and validation

**Figure 3:**
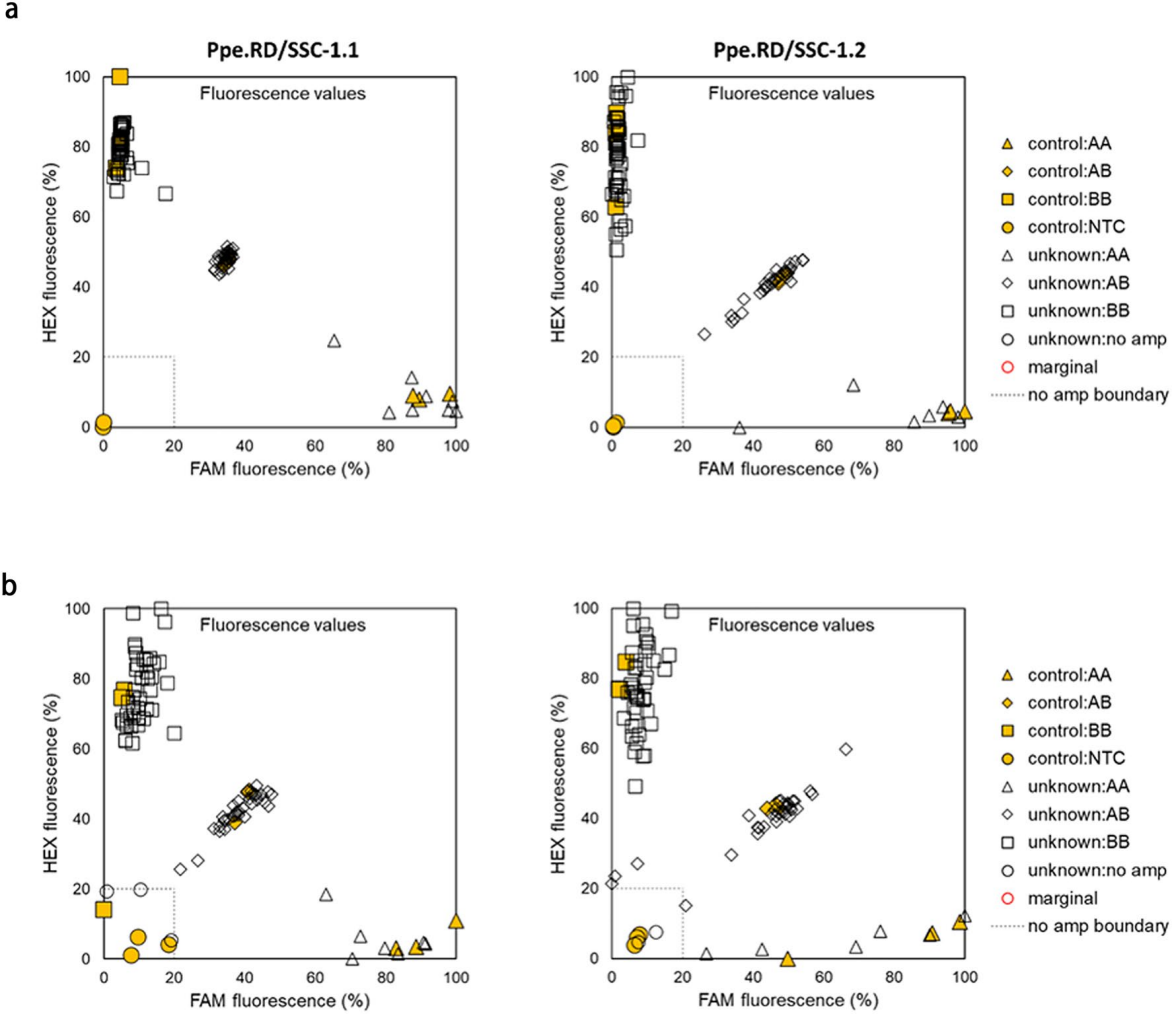
*Ppe.RPT/SSC* KASP assays validation in 84 peach samples using real-time (**a**) and endpoint (**b**) PCRs. Yellow triangles, diamonds, squares, and circles indicate genotypes AA, AB, BB, and non-template controls, respectively.

In order to confirm the predictive SNP and reconstruct haplotypes (possible six and observed four), all three SNP markers (SNP_IGA_422637, SNP_IGA_412338 and SNP_IGA_412662) were used in the KASP assay development and validation. The two KASP assays, Ppe.RPT/SSC-1.1 (SNP_IGA_411637) and Ppe.RPT/SSC-1.2 (SNP_IGA_412338), were developed and successfully amplified and distinguished alleles in validation material (Table 3, Fig. 3). The SNP_IGA_412662 was unsuitable for conversion into a KASP assay, due to its flanking sequence not meeting criteria for primer design, and was subsequently discarded. Using 84 peach cultivars, the assay validation revealed four different clusters corresponding to each genotype (AA, AB, and BB) and non-template controls (Fig. 3, Supplementary Table S8). The KASP had 93.1% accuracy in correct allele assignment compared to the 9K SNP array, with the discrepancies observed in four cultivars (China Pearl, Fantasia, Le Grand, and Saturn) (Supplementary Table S8). Additionally, end-point PCR for the same 84 peach cultivars showed similar performance to real-time PCR (Fig. 3), with only 3.2% samples needing to be repeated due to the lack of amplification.

**Figure 4:**
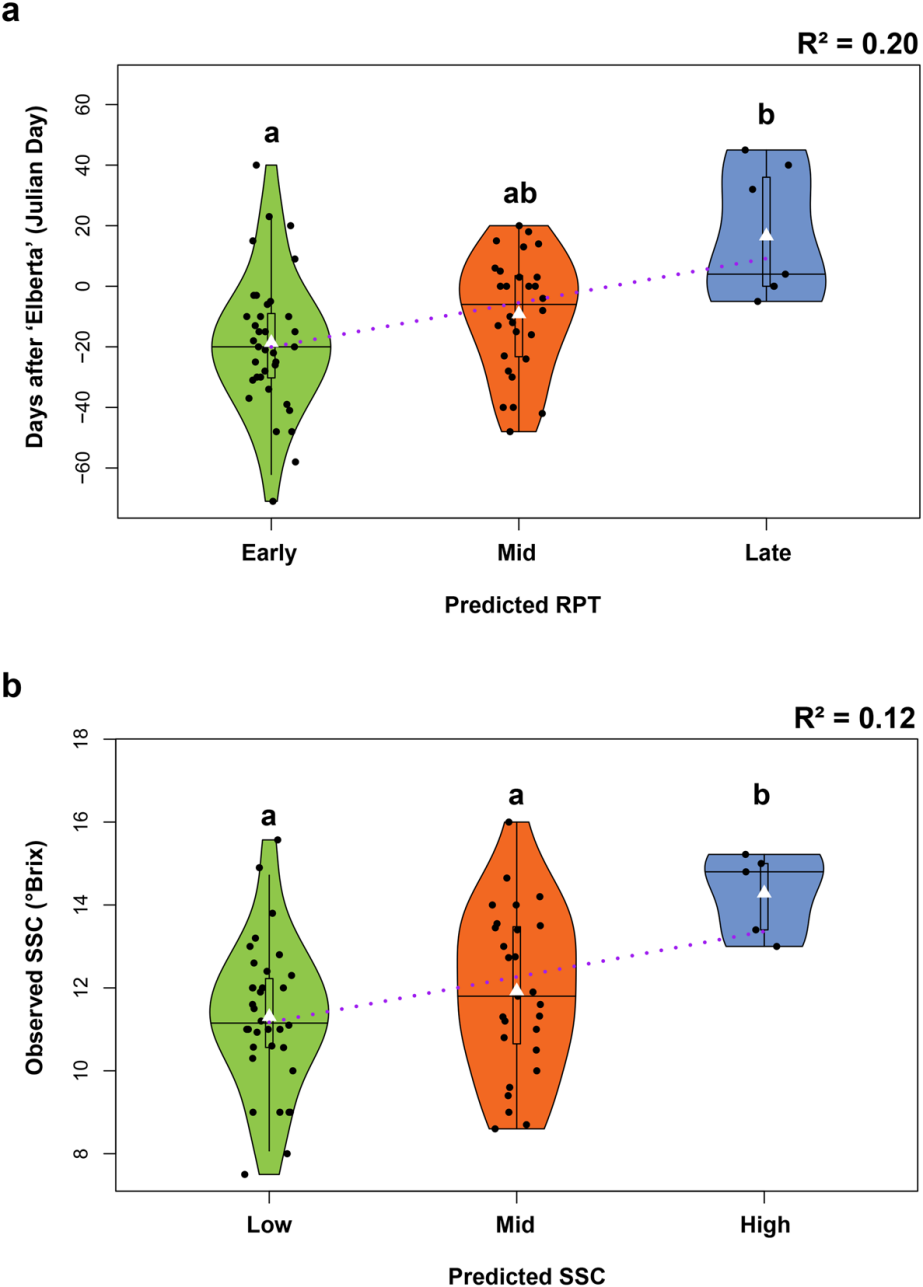
Comparison of the predicted and observed phenotypes for ripening time (RPT) (**a**) and soluble solid concentration (SSC) (**b**) in 84 peach cultivars used for assay development and validation. Phenotypic means that were significantly different (Tukey, *p* < 0.05) are represented by different letters. White triangles represent mean phenotypic values. Black horizontal lines represent median phenotypic values.

**Figure 5:**
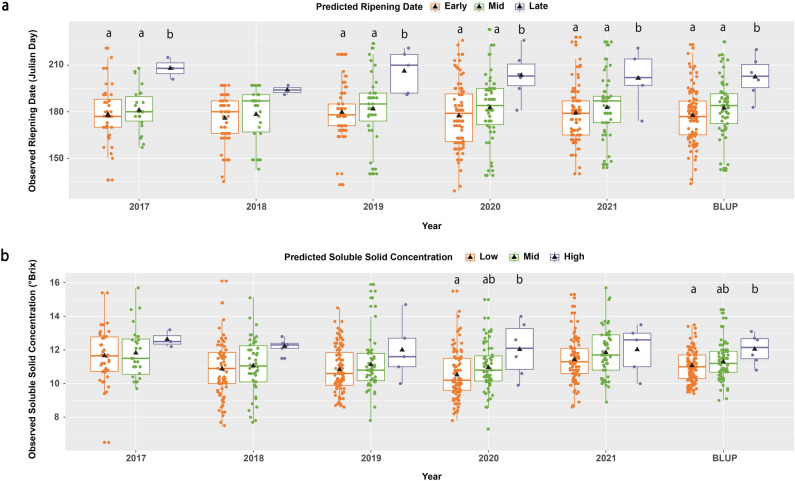
Comparison of the predicted and observed phenotypes for ripening time (**a**) and soluble solid concentration (**b**) in the Clemson University peach breeding program’s breeding materials across five different seasons and BLUP values. Phenotypic means that were significantly different (Tukey test, p < 0.05) are represented by different letters. Black triangles represent mean observed trait values, and horizontal lines represent median observed trait values.

Similarly, to the haplotype phenotype analyses above, the analysis of the phenotypic effect of the SNP_IGA_411637 (*Ppe.RPT/SSC-1.1*) in the 84 peach cultivars suggested that the ‘A’ allele was associated with a delay of RPT and an increase of SSC, while the ‘B’ allele was related to the advancement of RPT and a decrease of SSC. The comparison between observed and predicted mean RPT (early, mid and late) in the validation material (84 cultivars) explained 20% of the phenotypic variance observed and revealed a significant difference (Tukey test, *p* < 0.05) between early and late-ripening individuals (Fig. 4a). Individuals predicted as early and mid-season ripened on average 18 and 9 days before ‘Elberta’, respectively (Supplementary Table S9). In contrast, individuals predicted as late season had, on average, a delay of 16 days compared to ‘Elberta’ (Supplementary Table S9). The comparison between observed and predicted mean of SSC, showed that 12% of phenotypic variance can be explained by *Ppe.RPT/SSC-1.1*. Individuals with low and mid predicted SSC revealed a significantly (Turkey, *p* < 0.05) lower mean of SSC when compared with individuals with high predicted SSC (Fig. 4b). No significant differences were observed between individuals with low and mid predicted SSC; however, individuals predicted to have low SSC exhibited lower observed SSC levels than those with mid predicted SSC. The same patterns were observed for the 163 individuals from the CUPBP and 26 commercial cultivars with known RPT and SSC in five evaluated seasons. In these materials, individuals with the AA genotype (predicted to have late RPT and high SSC) had the latest average RPT and highest average SSC in all five seasons (Fig. 5). These individuals ripened on average three and four weeks later than mid (AB genotype) and early (BB genotypes) season cultivars, respectively (Fig. 5, Supplementary Table S10). Regarding SSC, less significant differences according to Tukey test (p < 0.05) were observed in the CUPBP material between the three genotypes of *Ppe.RD/SSC-1.1*, with late-ripening individuals (genotype AA) having 1.0 and 0.6 °Brix more than early and mid-season cultivars, respectively (Fig. 5, Supplementary Table S10).

## Discussion

The phenotypic mean values of RPT observed in this study were higher than those reported by Rawandoozi et al.^17^ and Nuñez-Lillo et al.^13^ and in agreement with the mean values observed by Eduardo et al.^10^. On the other hand, the mean values of SSC reported in this study were slightly lower than the values described by Nuñez-Lillo et al.^12^ and similar to the mean values observed by Rawandoozi et al.^16^. The differences in phenotypic means for RPT and SSC, observed in this study compared to previous works, could be attributed to the different germplasm evaluated and the focus of the breeding program. For example, the dataset analyzed by Rawandoozi et al.^17^ corresponded to seedlings from seven F1 low to medium chill full-sib families, where the RPT occurs earlier compared to the seedlings analyzed in this study. We observed significant differences between seasons in the mean values of RPT and SSC. However, moderate (0.41) to high (0.90) correlation coefficients between seasons were recorded for SSC and RPT, respectively.

Pedigree based QTL mapping using FlexQTL™ enabled the successful mapping of stable QTLs with decisive evidence (BF > 10) for RPT and SSC. The *qRPT.1* and *qSSC_4.1* detected in both seasons in an overlapped genetic interval formed a QTL cluster on LG4 and confirmed the QTLs and associated signals identified in previous studies using different germplasm and approaches^11,13–15,18^. The RPT and SSC QTL cluster initially covered a genetic region from 44 to 49 cM (from 10,981,971 to 12,523,245 bp) and comprised eight SNPs that could be used for haplotype analysis. Recent genome re-sequencing and transcriptome analyses of this region revealed polymorphisms in two NAC genes (*PpNAC1* and *PpNAC5*) suggesting that they might be good candidates for controlling fruit ripening^35^. For *PpNAC1*, a nonsynonymous G/A SNP in the third exon, and for PpNAC5, a 9-bp insertion in the third exon, were suggested as the most likely causal mutations linked to differences in ripening times^12,35^. Thus, the genetic region was narrowed down to 44–45 cM (physical position from 10,981,971 to 11,298,736 bp) and three SNPs using the peak position of the RPT and SSC QTLs in the FlexQTL™ outputs in the average datasets. Average datasets have been frequently used for determining the genetic region for further haplotype analysis using FlexQTL™^16,17,36^. The target region comprised three SNPs and allowed the identification of QTL genotypes with different effects of increasing/decreasing RPT and SSC (Fig. 1, Table 3). In addition, a predictive marker (SNP_IGA_411637) distinguished the allele associated with increasing (A) and decreasing (B) of the RPT and SSC in the breeding populations. These findings suggested that the marker-assisted selection (MAS) with the SNP_IGA_411637 could efficiently be used for selecting parents/seedlings for targeted RPT and SSC.

Previous RPT and SSC QTL mapping in peach focused mainly on identifying marker-trait association with candidate genes analysis in the mapped QTL interval. In this study, our primary goal was not only to map or validate QTLs, but also to convert the findings into a diagnostic DNA test for routine application in breeding. The complexity of RPT and SSC traits due to their polygenic nature and environmental influence^37^ made development of breeding tools for MAS difficult. However, quantitative traits were recently targeted in peach for DNA test development resulting in tools such as the *Ppe.CR.1* designed for predicting chilling requirement^24^. RPT and SSC display similar patterns to chilling requirements, where multiple genes control the phenotype and the major QTL explained more than 30% of the phenotypic variation. Thus, the findings obtained from the QTL mapping and haplotype analysis performed in this study suggested that it was feasible to develop a DNA test for predicting RPT and SSC in peach. The *Ppe.RPT/SSC-1* was designed using a recently reported KASP approach for fruit response to *Xanthomonas arboricola* pv. *pruni*^23^ and chilling requirement^24^ prediction in peach. In combination with crude DNA extraction^38^, this technology provides an inexpensive, trustworthy, and user-friendly tool for accurately predicting SNP genotypes^29,39^.

The validation of the *Ppe.RPT/SSC-1* breeding tool revealed concurrence between the outcomes of real-time and endpoint PCRs, indicating that endpoint PCRs can be utilized to improve throughput. Additionally, the *Ppe.RPT/SSC-1* assay developed for SNP discrimination successfully predicted the genotypes previously obtained from the 9K SNP array with disagreement in only four accessions. From those, two accessions (‘Fantasia’ and ‘Saturn’) have already demonstrated discrepancies between the SNP array and KASP genotypes in validation of *Ppe.CR.1* for prediction of chilling requirement^24^. This result supports the hypothesis that the discrepancies could be due to the incorrectly called array genotypes or different DNA sources used in the array (genotyping carried out by the RosBREED project^22^) and KASP genotyping. However, the peach array is no longer available for genotyping, making it impossible to genotype this material and identify the real cause of the discrepancy. Nevertheless, name ‘Saturn’ is often used to refer to flat peaches in general, which suggests high possibility of two different flat peaches carrying the same name being genotyped with the SNP array and the KASP assay.

Although two KASP assays were developed to distinguish haplotypes in the QTL target region (10,981,971 bp to 11,298,736 bp), only one (*Ppe.RPT/SSC-1.1*) was needed to distinguish the early and late-season ripening cultivars, confirming the results obtained from the haplotype analysis that indicated the SNP_IGA_411637 as a predictive marker. The predictive ability of *Ppe.RPT/SSC-1.1* for RPT of materials from different genetic backgrounds and environments (AR, SC, and TX) highlighted the usefulness of this tool for breeding. Moderate to high correlation between RPT and SSC were detected in this work. However, the predictive ability of *Ppe.RPT/SSC-1* was lower for SSC in comparison to RPT. Possible explanations for the lower prediction values could be due to the majority of the QTL effect observed in the SSC being attributed to RPT. This hypothesis was confirmed by the SSC QTLs runs where the RPT was used as cofactor and resulted in a lower BF (evidence of the presence of QTL) and the PVE. Effect of other QTL regions on different chromosomes associated with the genetic control of SSC^13,15,18,40^ could also affect prediction. For example, the major QTL for SSC was mapped on the chromosome 5^16^ in the Texas material, where the lowest prediction of SSC was observed. The *Ppe.RPT/SSC-1.1* developed in this study, did not account for other regions, which could explain the lower SSC prediction when compared to RPT. Lastly, the strong influence of environmental conditions on the final sugar content^37^ or the possibility that fruits in different ripening stages (under-ripened) could have skewed SSC values as only five fruits were considered for juice extraction and SSC determination. Thus, further studies focusing on DNA tool development accounting for other chromosomal regions associated with SSC and the appropriate ripening stage of the fruits selected for °Brix determination are required to obtain a high predictive accuracy for SSC.

## Conclusion

We successfully mapped/validated a stable QTL cluster on LG4 associated with RPT and SSC using a pedigree-connected germplasm comprising 288 individuals. In addition, we identified statistically different QTL genotypes in the target genetic interval selected for haplotype analysis. We also developed an accurate KASP assay for RPT and SSC identifying the correct genotype for 93% of samples with known genotypes and validated the assay on individuals from different genetic backgrounds and environments (AR, SC and TX). Furthermore, a prediction accuracy of the KASP assay and an ability to distinguish between early and late-season ripening and low and high SSC cultivars was obtained. The low-cost and quick crude DNA extraction combined with the KASP approach reported here provide relevant information to breeders for large-scale genotyping in breeding programs, saving time, and resources and efficiently accelerating the selection of desired individuals. Given the narrow genetic base of the peach germplasm and the fact that majority of modern peach breeding programs in the world share the same few cultivars in the pedigree of their breeding germplasm, suggest high relevance and utility of the KAPS assay developed in this study to peach breeding programs in the US and world. The results of this work will be extremely helpful for strengthening the bridge between academic research and breeding applications and, consequently, for peach genetic improvement.

## Methods

### Plant material

The material used in this study was comprised of pedigree connected germplasm with cultivars, advanced selections, and seedlings from Clemson University peach breeding program (CUPBP), which were previously assembled under the RosBREED project^18,22^. A breeding population containing a total of 288 seedlings, which included multiple F_1_ and F_2_ families obtained from 19 parents (Supplementary Table S1), was chosen for mapping analyses. The parents and seedlings were maintained at the Clemson University Musser Fruit Research Center, in Seneca, South Carolina (Latitude: 34.639038, Longitude: − 82.935244, Altitude 210 msl), under warm, humid, temperate climate and standard commercial practices for irrigation, fertilization, and pest and disease control. The trees were grafted on ‘Guardian®’ rootstock, and either planted at 4 × 6 m and trained to open center or at 1.5 × 4 m and trained to perpendicular V.

### Phenotypic data

Phenotypic data for QTL mapping and DNA test development were recorded over two seasons (2011–2012). Ripening time (RPT), in Julian days (JD), was determined when 20% of fruits were at commercial harvest by visually inspecting for the presence of a few soft fruits in the field for maturity twice per week. Representative fruit sample from each accession was harvested at the determined RPT. Juice was extracted from a composite sample, comprised of one approximately 2 cm wide longitudinal slice from each of five fruits, and SSC (°Brix) was measured using a digital refractometer. Statistical analysis (descriptive, normality test, Spearman’s rank correlation, and Wilcoxon signed rank test) was performed using R Statistical Software (version 4.1.2).

The narrow-sense heritability (h^2^) was estimated considering the two seasons (2011–2012) using the R package Sommer^41^ and the *vpredict* function:

$$vpredict\left(object, transform\right),$$

where object represents a model fitted with the mmer function; transform is the formula to calculate the function.

$$mix<-mmer\left(Trait\sim Year,random=\sim vsr\left(Selection,Gu=K\right),rcov=\sim vsr\left(dsr\left(Year\right),units\right),data=Trait\right),$$

where “K” refers to the additive relationship matrix. The formula included in the function was:

$${h}^{2}=\frac{VA}{VP},$$

where “VA” is the additive genetic variance; “VP” is the phenotypic variance.

### Genotyping and linkage map

Samples were genotyped with the IPSC peach 9K SNP array v1^33^. The SNP data curation was performed using the workflow for high-resolution genetic marker data described in Vanderzande et al.^21^. A total of 4005 SNPs was retained after the data curation process. Following linkage disequilibrium analysis and removal of redundant SNPs, a total of 1487 informative SNPs (Supplementary Table S4) were considered for building the linkage map used in the pedigree-based QTL mapping in FlexQTL™ software. The genetic positions of the retained SNPs were calculated using the physical position of the peach reference genome v2.0^34^ and a conversion factor where every 1 Mb corresponded to 4 cM, as described by Vanderzande et al.^21^.

### QTL mapping

The QTL mapping was performed using FlexQTL™ software (version 0.1.0.42)^42^ which implements pedigree-based QTL analysis via Markov Chain Monte Carlo (MCMC) simulation. The analysis was run at least twice for both seasons (2011 and 2012), as well as for the datasets RPT_Ave and SSC_Ave (average of the two seasons), until reaching the effective chain size (ECS) criterion for convergence (≥ 100). The MCMC length for RPT ranged from 700,000 to 900,000 iterations to store 1000 samples with a thinning between 700 and 900 under mixed genetic model. However, 200,000 iterations with a thinning of 200 under additive genetic model were adequate to achieve ECS ≥ 100 for SSC. Inference on the number of QTLs was based on a pairwise comparison of models (1/0, 2/1, 3/2, and so on) using twice the natural log of the Bayes factor (2lnBF) statistic. The Bayes factor (BF) parameter was interpreted as: non-significant (0–2), positive (2–5), strong (5–10), or decisive (> 10) evidence for the presence of QTLs^43^.

In this study, we reported only stable QTLs. A QTL was considered “stable” when: the BF was decisive (> 10) and the significant effect was located in overlapping positions between the two seasons. The stable QTLs were named as “*q*” + trait name abbreviation + data set + scaffold + number of the chronological QTL for this trait reported on this chromosome (e.g. *qRPT_SC_4.1*).

To narrow down and re-define the QTL intervals, further analysis was carried out in FlexQTL™ using the ‘MQTRegions.new’ file considering the supporting data files ‘Post_genome.csv’ and ‘marker map’. The newly generated output files were used to recalculate the phenotypic variance explained (PVE) for the stable QTLs and to update information for QTL intensity, interval, and mode positions.

FlexQTL™ obtained the additive variance (σ^2^_A(trt)_) for each trait by subtracting the residual variance (σ^2^_e_) from the phenotypic variance (σ^2^_P_). The phenotypic variance explained (PVE) for a particular QTL considering an additive model was calculated using the following equation:

$$PVE \, additive \, model= \frac{{\sigma }_{A\left(qtl\right)}^{2}}{{\sigma }_{P}^{2}}\times 100,$$

where ${\sigma }_{A\left(qtl\right)}^{2}$ is the additive variance of QTL.

Regarding the mixed model, genetic variance (${\sigma }_{G}^{2}$), was calculated by subtracting the residual variance (${\sigma }_{e}^{2}$), from the phenotypic variance (${\sigma }_{P}^{2}$), and the PVE was calculated as follows:

$$PVE \, mixed \, model= \frac{{\sigma }_{A\left(qtl\right)}^{2}+ {\sigma }_{D\left(qtl\right)}^{2}}{{\sigma }_{P}^{2}}\times 100,$$

where $${\sigma }_{A\left(qtl\right)}^{2}\text{ is the additive variance of QTL}$$ and $${\sigma }_{D\left(qtl\right)}^{2}\text{ is the dominant variance of QTL}.$$

A pleiotropic effect of RPT and other quality traits, including SSC, has been previously reported^11^. Therefore, QTL analysis for SSC_Ave (average of the two seasons) was also performed using the RPT information as a covariate, which was integrated as a nuisance variable in the data file used in FlexQTL™. Separate runs using a mixed model, with the same number of seeds and MCMC length (1,200,000), were carried out in FlexQTL™ using the RPT information in four different datasets: 1. Dataset where no covariate was included; 2. Dataset where the corresponding phenotypic average of RPT (in Julian days) was included as a cofactor; 3. Dataset where the haplotype information of the RPT QTL interval was included as a cofactor; 4. Dataset where both the phenotypic average of RPT (in Julian days) and haplotype information of the RPT QTL interval were included as cofactors.

### Haplotype analysis and predictive SNP

As described by Rawandoozi et al.^16^, the haplotype analysis was carried out by selecting SNPs within the significant QTL interval considering the RPT and SSC average datasets. We used the output files ‘MQTRegionsGTP.csv’ and ‘mhaplotypes.csv’ generated by FlexQTL™ to perform the haplotype analysis. Haplotypes were constructed in the dataset using PediHaplotyper R package^44^. The effects were determined from combinations of diplotypes. The nonparametric multiple comparison Steele–Dwass test (*p* < 0.05) was applied to assess significant differences between diplotype effects. QTL allele genotypes (*Q* or *q*) were assigned to haplotypes based on the direction of their effects (increasing or decreasing RPT and SSC, respectively). The statistical analysis was performed using the JMP Pro Version 13.2 (SAS Institute Inc., Cary, NC, 2016). Illumina codes for nucleotides, where A = A or T, and B = C or G, were used as marker designation in haplotypes^34^.

Phenotypic variation explained (PVE) by each informative SNP within the significant QTL interval was estimated using single linear regression (SLR) analysis in R Statistical Software (version 4.1.2). The phenotypic performance of different genotypes of the predictive marker was further validated in peach breeding populations comprising 128, 290 and 139 individuals from Arkansas (AR), South Carolina (SC) and Texas (TX), respectively.

### KASP marker development and validation

Two informative SNPs capable of distinguishing haplotypes for the main RPT and SSC QTL on LG4 (10,981,971 to 11,298,736 bp) were used for developing the *Ppe.RPT/SSC-1* KASP assay. The SNP_IGA_412662 was unsuitable for conversion into a KASP assay due to its flanking sequence not meeting the criteria for primer design and was subsequently discarded. Primers were designed for each of the two SNPs (Table 3) and reaction mixtures and PCR conditions for the KASP assays were determined following Fleming et al.^22^. Eighty-four peach cultivars representing the diversity of fresh-market US germplasm, 51 of which had known genotypes from the 9K SNP array^33^, were chosen for the development and validation of the assay (Supplementary Table S3). Phenotypic data for RPT were obtained from literature^45^, while the SSC data were obtained from two databases: Clemson University Variety evaluations database (https://www.clemsonpeach.org/), and the Genome Database for Rosaceae^46^. The variety evaluation data from Musser fruit research farm, were collected within 2015–2021, and the publicly available GDR peach data (GRIN_PEACH and Peach RosBREED Public), were collected during three and seven seasons, within RosBREED^22^ project and GRIN, respectively. The average SSC for each cultivar was calculated and used in the validation of the assay. DNA for these cultivars was extracted using the protocol described in Edge-Garza et al.^38^.

Three replicates of non-template controls and positive controls for homozygous (AA and BB) and heterozygous (AB) genotypes were tested in a 96 well-plate for each assay. Positive controls were selected from the DNA samples with known genotypes from the 9K SNP array (Supplementary Table S11). Amplifications were conducted in a Bio-Rad CFX Connect Real-Time PCR thermocycler under a standard protocol (15 min at 94 °C; 10 cycles of 20 s at 94 °C and 60 s at 61 °C with a 0.6 °C decrease in temperature per cycle; 40 cycles at 94 °C for 20 s; 60 s at 55 °C, and 30 s at 23 °C), and cycle 25 of real-time PCR was selected for each KASP assay to maximize separation between genotypes. A template spreadsheet designed by Fleming et al.^23^ was used to assign the genotypes to each sample based on the relative fluorescence unit values from this cycle. For routine use, end-point PCR was tested on Bio-Rad T100 thermocycler using the same cultivars and positive controls for all assays, with slightly modified protocol. The number of cycles at the SNP specific temperature was decreased to 25 and the last cycle of the standard protocol (30 s at 23 °C) and plate reading were omitted. End-point reactions were read on the Bio-Rad CFX Connect Real-Time PCR thermocycler using Bio-Rad CFX Maestro™ software.

The newly developed *Ppe.RPT/SSC-1* KASP assays were validated by screening 163 seedlings from the CUPBP and 26 commercial cultivars (Supplementary Table S12) with end-point PCR. The validation set included 15 seedlings from QTL mapping germplasm. Phenotypic data was collected in the CUPB program. The best linear unbiased predictions (BLUPs) for each individual were obtained for the RPT and SSC values recorded across five seasons (2017 to 2021) using the R package ‘lme4’^47^ with year selected as a random effect:

$${Y}_{ij}= \mu +{g}_{i}+ {y}_{j}+ {gy}_{ij} + \varepsilon ,$$

where *Y*_*ij*_ is the trait of interest, *µ* is the overall mean, *g*_*i*_ is the genetic effect of *i*th genotype, *y*_*j*_ is the effect of the *j*th year, and *gy*_*ij*_ as the interaction effect of *i*th genotype with *j*th year, *ɛ* is the residual of the model.

DNA samples for these individuals were obtained using a rapid crude DNA extraction protocol described by Noh et al.^48^. The template spreadsheet designed by Fleming et al.^23^ was employed to automatically assign genotypes. Statistical differences between classes were evaluated using one-way ANOVA, followed by the Tukey test for multiple comparisons in SPSS.

### Experiments involving plants

Relevant guidelines and regulations have been followed for all experiments involving plants. Plants subject to these experiments were created in the co-authors breeding programs and belong to them so the permission or license to use them is not needed.

### Supplementary Information

Supplementary Figures.

Supplementary Tables.

## Data Availability

The datasets presented in this study can be found in online repositories. The names of the repository/repositories and accession number(s) can be found below: www.rosaceae.org.
