# Supplementary material for: Ppe.RPT/SSC-1: from QTL mapping to a predictive KASP test for ripening time and soluble solids concentration in peach
Source: Sci Rep. 2024 Jan 17;14:1453. doi: 10.1038/s41598-024-51599-2 (PMC10791670; doi:10.1038/s41598-024-51599-2)
Supplement: Supplementary file 1 — Supplementary Figures. [file 41598_2024_51599_MOESM1_ESM.pdf]

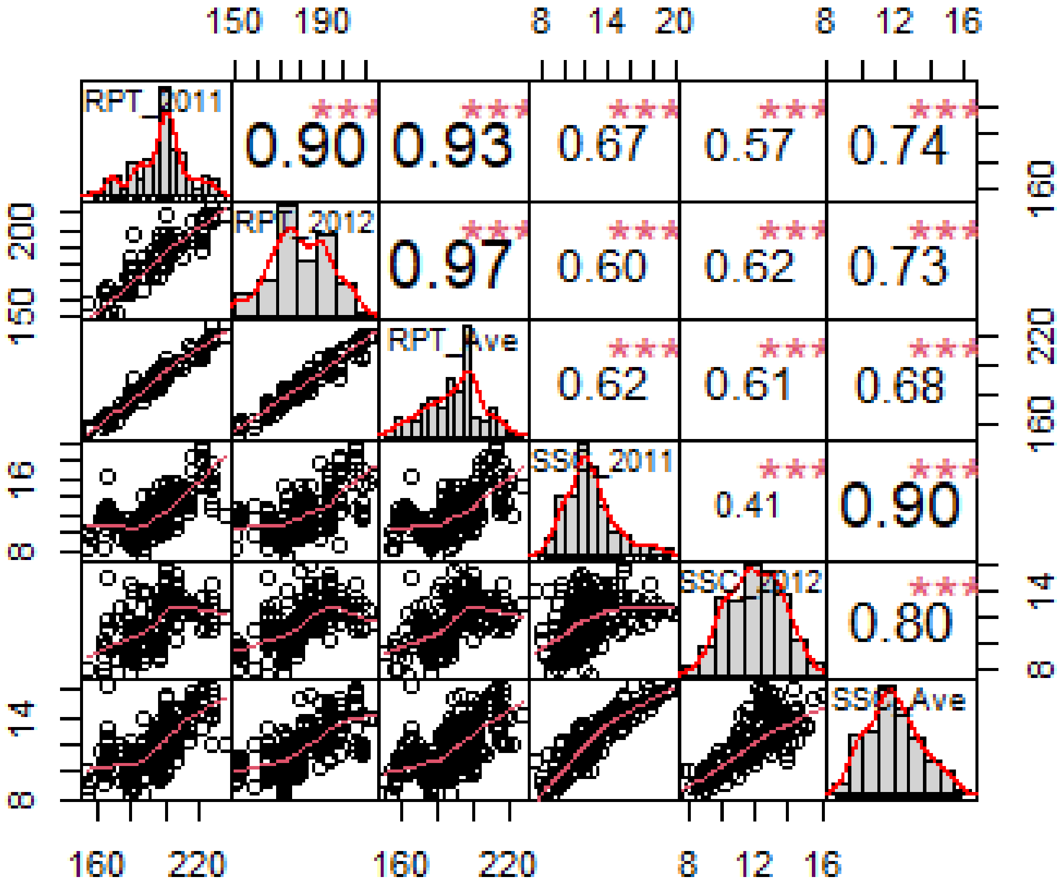

Supplementary Fig. S1: Supplementary Fig. S1: Distribution of ripening time (RPT and SSC (on the diagonal); bivariate scatter plots with a fitted line (on the bottom of the diagonal); correlations plus the significance level of traits and seasons in the evaluated 288 peach seedlings from Clemson University peach breeding program (CUPBP) in 2011, 2012 and average datasets (on the top of the diagonal).

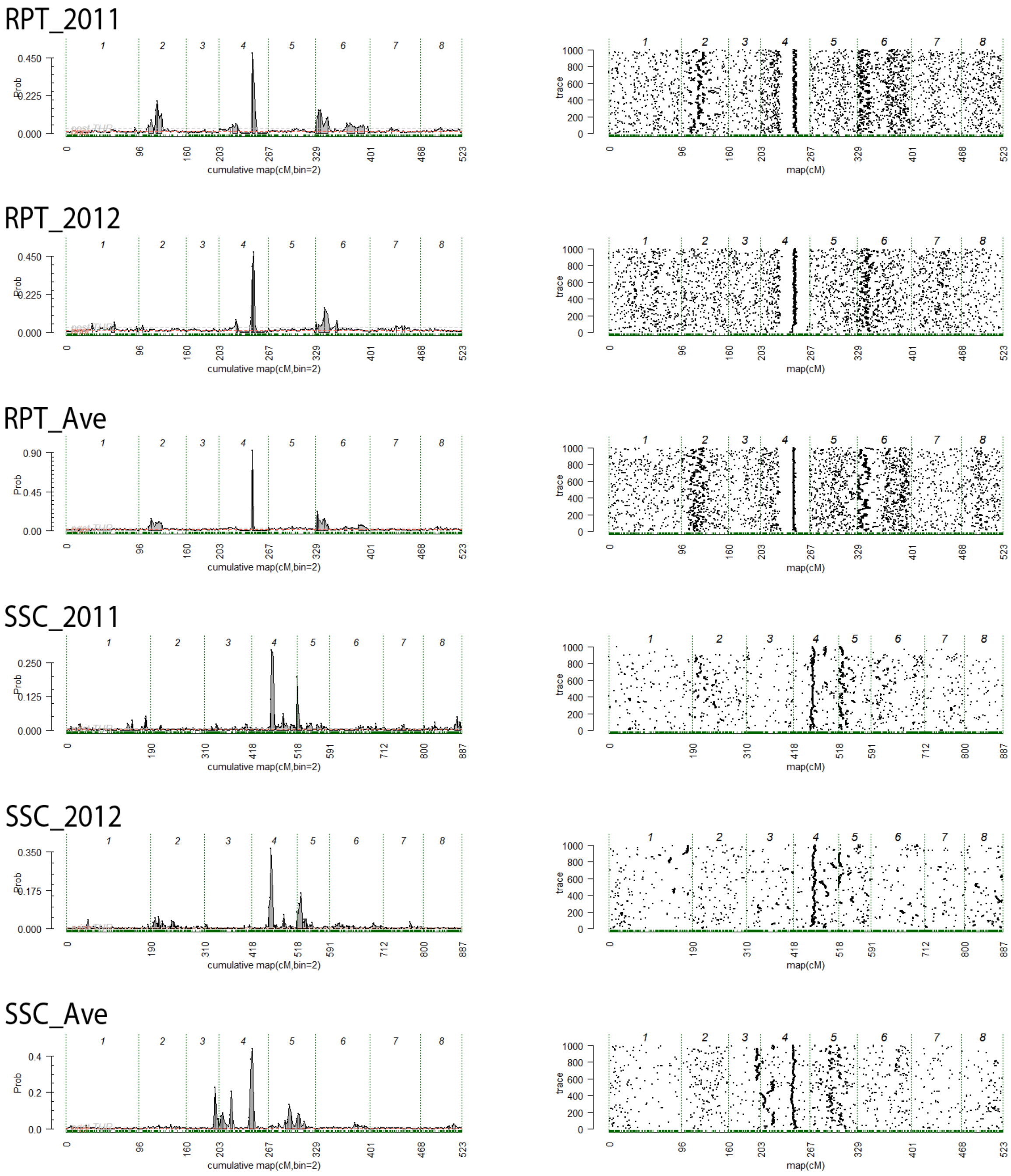

Supplementary Fig. S2: Supplementary Fig. S2: Posterior positions (left) and trace samples QTL positions (right) based on mixed (RPT) and additive (SSC) model performed using Visual FlexQTL software for peach RPT and SSC from Clemson University peach breeding program in two seasons (2012, 2013) and average datasets.

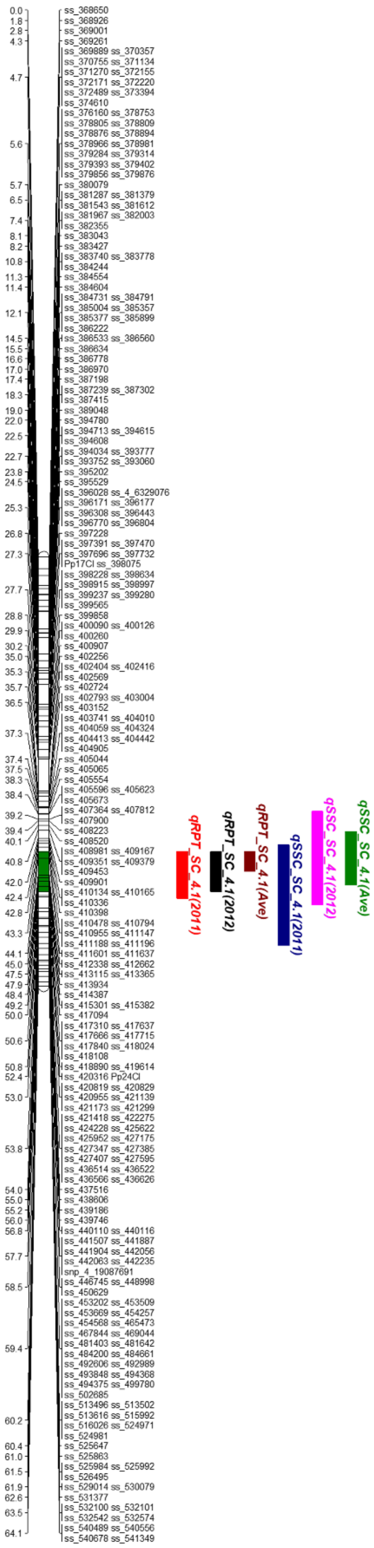

Supplementary Fig. S3: Stable QTLs controlling ripening time (RPT) and soluble solids concentration (SSC) on LG4 in peach. Marker names are listed at the right side, and the genetic distances (in centimorgans) are listed at the left. QTLs are drawn at the right of each LG. QTLs for RPT and SSC are represented with different bar fills: red, black and brown for RPT, blue, pink and green for SSC.
